# Pathways for Patients with Transthyretin Amyloid Cardiomyopathy from a District General Hospital Perspective

**DOI:** 10.3390/jcdd13060248

**Published:** 2026-06-04

**Authors:** Chun Shing Kwok, Pippa Hamnett, Matt Palmer, Dennis Chong

**Affiliations:** 1 Department of Cardiology, Mid Cheshire Hospitals NHS Foundation Trust, Crewe CW1 4QJ, UK; pippa.hamnett@mcht.nhs.uk (P.H.); dennis.chong@mcht.nhs.uk (D.C.); 2 Digital Technology & Information Services, Mid Cheshire Hospitals NHS Foundation Trust, Crewe CW1 4QJ, UK; matt.palmer@mcht.nhs.uk

**Keywords:** transthyretin amyloid cardiomyopathy, patient pathways, diagnosis

## Abstract

Background: The care of patients with transthyretin amyloid cardiomyopathy (ATTR-CM) is often fragmented and routine datasets rarely capture real-world clinical trajectories and reasons for diagnosis. We introduce a novel approach, called forensic data acquisition and pathway analysis, to examine the real-world experiences of patients with ATTR-CM in our district general hospital. Methods: We retrospectively evaluated inpatient and outpatient healthcare records for our hospital between 2019 to 2025 as a part of a quality improvement project. Results: We identified 26 cases of confirmed or likely wild-type ATTR-CM and four hereditary cases from two families carrying the S77Y variant and estimate the prevalence of transthyretin cardiac amyloidosis to be 1 per 10,000 patients. Many red flags were present in patients, including carpal tunnel syndrome (63.3%) and lumbar spinal stenosis (26.7%), as well as echocardiographic features of left ventricular hypertrophy (86.7%), left atrial dilatation (76.7%), right ventricular hypertrophy (43.3%), and a dense or speckled myocardial appearance (43.3%). Among patients with wild-type disease, the most frequent trigger for further investigation was the presence of suspicious features on transthoracic echocardiography, accounting for 13 cases. Incidental abnormalities detected on cardiac MRI contributed to another six diagnoses. In two patients, non-invasive imaging did not provide sufficient diagnostic certainty, and myocardial biopsy was required to confirm ATTR-CM. Conclusions: Forensic data acquisition and pathway analysis provides a powerful approach for revealing real-world clinical activity in ATTR-CM, exposing diagnostic patterns and missed opportunities that remain hidden in routine datasets.

## 1. Introduction

The rapid expansion of therapeutic options for transthyretin amyloid cardiomyopathy (ATTR-CM) has transformed the condition from a progressive and palliative disease into one where early detection and intervention can meaningfully alter outcomes. The clinical priority has shifted towards identifying patients who could benefit from treatment as early as possible. This is particularly important in ATTR-CM, where individuals often interact with hospital services frequently, as a study suggests that there is a median of 17 encounters in the three years preceding diagnosis [1]. However, mapping how patients move through the healthcare system remains challenging. Real-world pathways are shaped not only by clinical events but also by patient choices, clinician reasoning, and the natural progression of a disease that often evolves silently for years [2]. Moreover, the pathways may be better established in specialist centres with expertise and availability of investigations, and the activities that take place outside these settings is not well understood.

The concept of the patient pathway, first introduced in the patient pathway review [3], reframes traditional data-science variables as a sequence of clinical and non-clinical events unfolding over time. However, a fundamental limitation remains that these pathway reviews are largely theoretical.

We introduce a novel approach, called forensic data acquisition and pathway analysis, to examine the real-world experiences of patients with ATTR-CM in our district general hospital. Mapping patient events with a focus on high-quality data about clinically relevant events over time offers a powerful means to understand real-world care, diagnostic delays, and identify potential actionable gaps. This approach may help clinicians working outside or far from referral centres to better manage people living with ATTR-CM and improve their outcomes.

## 2. Materials and Methods

### 2.1. Study Setting and Design

This retrospective observational study is a health service evaluation that was designed to improve the quality of care for patients with ATTR-CM. This project was approved by our clinical effectiveness group.

Mid Cheshire Hospitals NHS Foundation Trust is a district general hospital which provides hospital care for a population of approximately 300,000 people living in the Cheshire area. Care for patients at the Trust is divided into the emergency department, inpatient services, outpatient services, and community services.

Forensic data acquisition involved detailed review of all clinical records over time for each patient for clinically relevant events. For each patient, activities relevant to the diagnosis and treatment of patients with ATTR-CM were captured over time and listed chronologically, unlike traditional data collection where all variables for data collection are prespecified and the data is either gathered if available or missing. The process of data acquisition had the flexibility to capture factors which may be important that are not pre-specified. This data-driven approach enables discovery of potentially important events in real-world settings. The data collector, therefore, needed to be an expert in the field to determine what is relevant to the management of patients with the condition, but was also required to have some understanding of data science to collect the data in a way that could be used in analysis.

Pathway analysis was then performed on the data to provide a simplified version of the key events. This method not only enabled us to capture the events that occurred but also potentially the reason for their occurrence.

### 2.2. Patient Identification

We reviewed paper-based and electronic hospital patient records of all patients with ATTR-CM. Inpatients with a diagnosis of ATTR-CM were initially identified from searching the secondary uses services (SUS) dataset for our hospital between 2019 to 2025 for the International Classification of Disease Tenth Revision (ICD-10) codes E85 (Amyloidosis). Our electronic outpatient dataset was also searched for any document with the string “amyloid” in the body of text. The full healthcare records for patients identified from inpatient and outpatient records were then reviewed to confirm that the diagnosis of ATTR-CM was correct.

### 2.3. Data Collected

For the patients that were identified to have ATTR-CM, the data collected included:

1. Care settings
2. Red flags for ATTR-CM such as left ventricular hypertrophy, carpel tunnel syndrome, lumbar spinal stenosis, and biceps tendon rupture [4].
3. Key symptoms such as breathlessness or chest pain.
4. Tests results such as echocardiography, DPD scan, cardiac MRI scan, and myocardial biopsy
5. Treatment.
6. Outcomes.

All events were then presented chronologically so that upstream and downstream events could be appreciated for each activity and how they fit into the patient pathway. Review of each patient pathway enabled highlighting any learning points from each case.

### 2.4. Data Analysis

Statistical analysis was performed on Stata 13.0 (College Station, TX, USA). Considering our population size, we estimated the prevalence of diagnosed ATTR-CM. Descriptive statistics are presented for each variable, with number and percentage for variables and mean and standard deviation for age at diagnosis of ATTR-CM. We also graphically present two of the patient pathways data pictorially. We explore the reasons for diagnosis for the population narratively based on the patient pathways.

## 3. Results

### 3.1. Prevalence of Diagnosed TTR Using Pathways Data

We identified 30 cases of transthyretin cardiac amyloidosis over the 5-year study period. Review of inpatient records identified eight patients, while 22 patients were identified from outpatient records. Considering that we have a population of 300,000 patients served by our district general hospital, we estimated the prevalence of transthyretin cardiac amyloidosis to be 1 case per 10,000 patients or about 1 case in 30,000 patients if deaths are removed. For wild-type ATTR-CM, this prevalence would be 1 case per 11,538 patients, and for hereditary ATTR with the S77Y variant, this prevalence would be 1 case per 75,000 patients.

### 3.2. Traditional Analysis to Evaluation of the Population

Table 1 summarises the descriptive characteristics of the ATTR-CM population. A total of 19 individuals (63.3%) received their diagnosis at the National Amyloidosis Centre (NAC), while nine patients (30.0%) died before they could be reviewed there, and two patients (6.7%) were never assessed at the NAC. The cohort included 26 cases of confirmed or likely wild-type ATTR-CM and 4 hereditary cases from two families carrying the S77Y variant. The population was predominantly male, with 28 men representing 93.3% of the total.

Red flags associated with cardiac amyloidosis were common. Carpal tunnel syndrome was present in 19 patients (63.3%), lumbar spinal stenosis in eight patients (26.7%), and biceps tendon rupture in two patients (6.7%). Atrial fibrillation was documented in 16 patients (53.3%), and erectile dysfunction in four patients (13.3%).

Echocardiographic assessment showed a spectrum of left ventricular systolic function: normal in 10 patients (33.3%), mildly impaired in 12 (40.0%), moderately impaired in four (13.3%), and severely impaired in another four (13.3%). The most frequent structural and functional abnormalities included left ventricular hypertrophy (26 patients, 86.7%), left atrial dilatation (23 patients, 76.7%), right ventricular hypertrophy (13 patients, 43.3%), a dense or speckled myocardial appearance (13 patients, 43.3%), pericardial effusion (12 patients, 40.0%), and aortic stenosis (two patients, 6.7%).

Diagnostic imaging was widely used. DPD scintigraphy was performed in 25 patients (83.3%) and cardiac MRI in 21 patients (70.0%). Two patients (6.7%) underwent bone scintigraphy and myocardial biopsy as part of their diagnostic work-up.

Regarding treatment, the most commonly prescribed therapy was tafamidis (10 patients, 33.3%), followed by vutrisiran (five patients, 16.7%), patisiran (four patients, 13.3%), and diflunisal (two patients, 6.7%). Overall, 19 patients (63.3%) in the cohort died with ATTR-CM, reflecting the severity of the disease and emphasising the importance of timely diagnosis and intervention.

### 3.3. Forensic Data Collection Presented in Pathway Form

**Figure 1:**
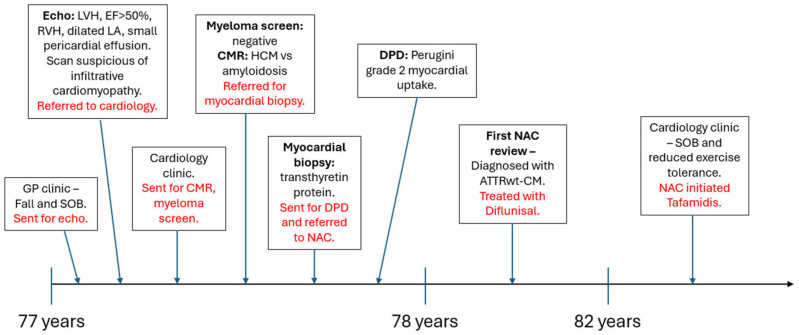
Graphical representation of the patient pathway for Case 1 (male) with wild-type ATTR-CM. Legend for Figure 1: GP = general practitioner, SOB = shortness of breath, LVH = left ventricular hypertrophy, RVH = right ventricular hypertrophy, LA = left atrium, CMR = cardiac magnetic resonance imaging, DPD = 3,3-diphosphono-1,2-propanodicarboxylic acid, NAC = National Amyloidosis Centre, ATTRwt-CM = wild-type transthyretin amyloid cardiomyopathy.

**Figure 2:**
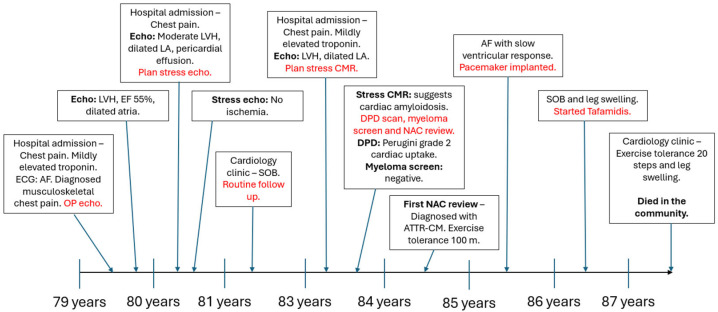
Graphical representation of the patient pathway for Case 2 (male) with wild-type ATTR-CM. Legend for Figure 2: AF = atrial fibrillation, OP = outpatient, LVH = left ventricular hypertrophy, LA = left atrium, CMR = cardiac magnetic resonance imaging, DPD = 3,3-diphosphono-1,2-propanodicarboxylic acid, NAC = National Amyloidosis Centre, SOB = shortness of breath, ATTRwt-CM = wild-type transthyretin amyloid cardiomyopathy.

The detailed findings from the forensic data collection were able to outline the clinical events and healthcare interactions for patients with wild-type transthyretin amyloid cardiomyopathy and hereditary transthyretin amyloidosis, respectively. Illustrative pathway reconstructions for Case 1 and Case 2 are shown in Figure 1 and Figure 2. These examples highlight the striking variability in real-world diagnostic trajectories. Case 1 demonstrates that under certain circumstances, care, diagnosis, and treatment can proceed rapidly and efficiently once key clinical features are recognised. In contrast, Case 2 illustrates how multiple opportunities for earlier diagnosis may be missed. Despite repeated hospital admissions for chest pain and echocardiographic features suggestive of cardiac amyloidosis, the diagnosis was only made incidentally during a stress cardiac MRI scan performed for another indication. Although tafamidis therapy was initiated after diagnosis, the patient died within a year, underscoring the consequences of delayed recognition and the narrow therapeutic window in which disease-modifying treatment is most effective.

### 3.4. Reasons for Diagnosis

**Figure 3:**
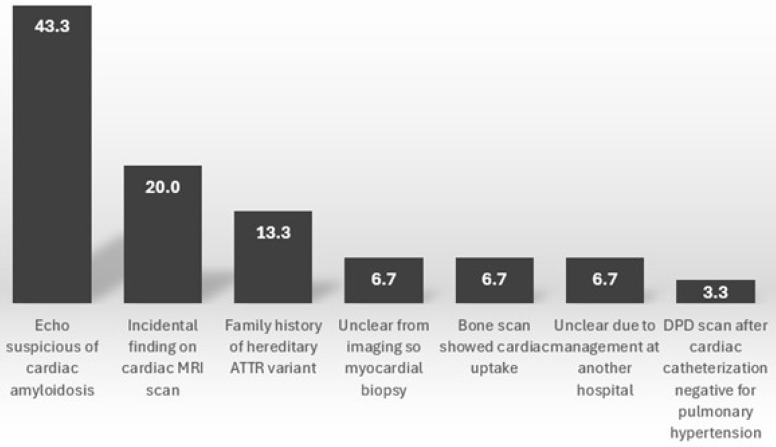
Reason for diagnosis of ATTR-CM. Legend for Figure 3: ATTR = transthyretin amyloidosis, MRI = magnetic resonance imaging, DPD = 3,3-diphosphono-1,2-propanodicarboxylic acid.

The reasons leading to a diagnosis of ATTR-CM are summarised in Figure 3. Among patients with wild-type disease, the most frequent trigger for further investigation was the presence of suspicious features on transthoracic echocardiography, accounting for 13 cases (43.3%). Incidental abnormalities detected on cardiac MRI contributed to another six diagnoses (20.0%), reflecting the growing role of advanced imaging in uncovering unrecognised amyloid cardiomyopathy. In two patients (6.7%), non-invasive imaging did not provide sufficient diagnostic certainty, and myocardial biopsy was required to confirm ATTR-CM. An additional two cases (6.7%) were identified when patients undergoing bone scintigraphy for prostate cancer were found to have unexpected myocardial tracer uptake. Finally, one patient (3.3%) initially evaluated for suspected pulmonary hypertension underwent DPD scintigraphy for unexplained breathlessness, which revealed findings consistent with cardiac amyloidosis.

All patients carrying the S77Y variant of familial amyloid polyneuropathy were identified through cascade screening after a relative was diagnosed. Each individual subsequently underwent genetic testing and was confirmed to carry the pathogenic variant. One patient initially presented with lower back pain and was diagnosed with lumbar spinal stenosis; notably, his brother also had lumbar spinal stenosis as well as carpal tunnel syndrome, both of which are recognised early manifestations of transthyretin amyloidosis. In a separate family, another patient first came to medical attention after experiencing a sudden loss of sensation while driving, and his brother had a history of bilateral carpal tunnel syndrome.

## 4. Discussion

In this manuscript, we introduce the concept of high-quality data collection using a pathway-based framework. By examining clinical activities in chronological sequence with a method termed forensic data acquisition, this approach not only documents what occurs during patient care but also clarifies why events unfold as they do. Such comprehensive evaluation supports traditional analytical methods while retaining the flexibility to detect unforeseen or emergent patterns within the data. The method of coupling the forensic data with pathway analysis further facilitates an appreciation of how upstream clinical events may generate downstream consequences, offering a more integrated understanding of patient trajectories. Using this method, we demonstrate how rigorous data collection and thoughtful data handling can illuminate the burden of cardiac amyloidosis, reveal the complexity of patient journeys, and highlight the insights that can be gained through an observational and data-driven approach.

The current report employs methodology that prioritise the collection of high-quality data. Data collection is an onerous aspect of research. While researchers with this task may receive training, the physical data collection may be undertaken by an individual who does not have experience with looking after patients with cardiac amyloidosis. In this study however, data collection was undertaken by a senior researcher, with expertise in both cardiology and data analytics. This approach ensures that clinically meaningful events are identified and recorded in a manner that enhances the interpretability of the dataset.

A further strength of the forensic data collection is its flexibility. Rather than prespecifying a fixed set of variables to collect, the data collector reviewed the complete patient records chronologically and documented key events relevant to the management of cardiac amyloidosis. This strategy is particularly important in the contemporary research environment, where artificial intelligence and data-mining techniques are increasingly used. For example, use of artificial intelligence-based search engines can help identify red flags that can substantially increase the detection of suspected ATTR-CM. However, these tools can only be used if electronic health records are available, which they were not in our district general hospital at the time of the evaluation. Moreover, if clinically significant data is not collected at the outset, no algorithm can infer their importance.

We adopted a comprehensive strategy to identify patients with cardiac amyloidosis. Using inpatient hospital billing data alone, only eight patients were captured through an ICD-10 diagnosis code for amyloidosis. An additional twenty-two cases were identified exclusively through detailed review of outpatient records. This discrepancy highlights major limitations of many contemporary datasets where outpatient encounters are frequently uncoded and not considered. Because outpatient care is typically reimbursed per consultation, the clinical activities associated with these visits, such as symptoms, examination findings, diagnoses, investigations, and treatments, are often not formally coded or structured. To address this gap, we manually reviewed outpatient documentation and searched for the term “amyloid” within free-text clinical notes. Given the relative rarity of the condition, this string-based search was feasible and enabled the identification of clinically relevant cases that would otherwise have been missed by relying solely on coded data.

This manuscript represents the first study to provide real-world data supporting the concept of patient pathways in cardiac amyloidosis [3]. In this work, we describe and quantify key real-world events, including misdiagnosis, failure to consider an underlying diagnosis, failure to order or correctly interpret diagnostic tests, and delays or omissions in referral to specialist services. Our data also demonstrates the diverse clinical settings in which patients with transthyretin cardiac amyloidosis may first present, such as heart failure clinics, incidental findings on echocardiography, bone scintigraphy or cardiac MRI, neurology clinics, and orthopaedic services. Although these findings are primarily generalisable to our district general hospital in the United Kingdom, they highlight a broader principle: any healthcare system aiming to deliver high-quality, aetiology-specific care for patients with heart failure should undertake similar pathway-based evaluations. Such analyses are essential for identifying gaps, understanding patient journeys, and improving diagnostic and referral processes.

Diagnostic delay is a key consideration when considering the pathways for patients with cardiac amyloidosis. Data from the National Amyloidosis Centre in the United Kingdom found that the median duration of associated symptoms before diagnosis fell from 36 months between 2002 to 2006 to 12 months between 2017 to 2021 [5]. A recent evaluation from the West German Amyloidosis centre suggests that the time to diagnosis for transthyretin amyloid cardiomyopathy decreased from 398 days to 277 days [6]. However, these findings are from specialist centres with dedicated teams to identify and treat the condition. Our current evaluation supports a narrow window of opportunity for patients, as patients with delayed diagnosis often die without confirmation of the diagnosis. Over the evaluation period, 63.3% of patients died (73.1% for wild-type ATTR-CM) and those who survived had younger age at diagnosis 71 ± 12 years compared (78 ± 6 years for wild-type ATTR-CM) to 80 ± 4 years for those that died.

The major clinical implication of this evaluation is the heterogeneity of care pathways for patients even at a single district general hospital. In the United Kingdom, treatment for transthyretin cardiac amyloidosis is highly centralised, with care coordinated through the National Amyloidosis Centre and supported by emerging satellite specialist centres in Birmingham and Liverpool. Within our local cardiology service, all consultants manage general cardiology patients, and a key conclusion is that practice could be improved through the development of a standard operating procedure and the designation of one or more consultants with a specific interest in amyloidosis to oversee patient pathways and facilitate rapid diagnosis. Echocardiography has a key role in the diagnosis and there are now guidelines for echocardiography [7], and specialised imaging techniques, including speckle-tracking echocardiography and measurement of global longitudinal strain, which also provide prognostic information [8].

This study has several limitations. The findings are generalisable to the cases of diagnosed ATTR-CM in our area and district general service. It is possible that there is undercapture of cases of undiagnosed cases of ATTR-CM due to under-recognition, genetic variability, and methodological bias. For example, search methodology could have been expanded to include diagnostic radiology databases but these were not available. In addition, care within the National Health Service is fragmented, meaning we cannot determine whether patients sought treatment at other hospitals, which contributes to missing data. Although our approach prioritised high-quality data collection and validation through cross-checking multiple data sources, it was both resource-intensive and time-consuming. Nevertheless, it generated rich, detailed information that enhances understanding of real-world clinical activity. While this method may not be suitable for large-scale data collection, it may be valuable for initial pilot studies to identify which data elements should be captured before undertaking larger investigations. Also, our study is limited because we did not have detailed electrographic data as the tracings were often lost from physical clinical notes. Finally, the retrospective design introduces the potential for recall bias. Ideally, clinical activities should be collected prospectively, which may be feasible for patients identified through genetic screening, but would be more challenging to prospectively monitor patients who may develop wild-type transthyretin cardiomyopathy.

## 5. Conclusions

We introduce the concept of forensic data acquisition and pathway analysis in ATTR-CM and estimate a prevalence of one case per 10,000 individuals in our population. Among patients with wild-type ATTR-CM, the most common trigger for further investigation was the presence of suspicious features on transthoracic echocardiography, with additional diagnoses arising from incidental abnormalities detected on cardiac MRI or bone scintigraphy. In contrast, all individuals carrying the S77Y variant of familial amyloid polyneuropathy were identified through cascade screening following a relative’s diagnosis. Overall, forensic data acquisition and pathway analysis provides a powerful approach for revealing real-world clinical activity in ATTR-CM, exposing diagnostic patterns and missed opportunities that remain hidden in routine datasets.

## Figures and Tables

**Table 1:** Characteristics, investigations, treatments, and mortality of patients with diagnosis of ATTR-CM.

Variable	Rate (%)
Diagnosis	
Confirmed at NAC	19 (63.3%)
Referred to NAC but died before review	9 (30.0%)
Not referred to NAC but likely diagnosis	2 (6.7%)
Type of ATTR	
Wild-type confirmed	15 (50.0%)
Likely wild-type	11 (36.7%)
Hereditary S77Y variant	4 (13.3%)
Male	28 (93.3%)
Female	2 (6.7%)
Clinical red flag or co-diagnoses	
Carpel tunnel syndrome	19 (63.3%)
Other peripheral neuropathy	6 (20.0%)
Lumbar spinal stenosis	8 (26.7%)
Biceps tendon rupture	2 (6.7%)
Erectile dysfunction	4 (13.3%)
Atrial fibrillation	16 (53.3%)
Echo left ventricular function	
Normal	10 (33.3%)
Mildly impaired	12 (40.0%)
Moderately impaired	4 (13.3%)
Severely impaired	4 (13.3%)
Echo findings	
Left ventricular hypertrophy	26 (86.7%)
Right ventricular hypertrophy	13 (43.3%)
Dense or speckle myocardium	13 (43.3%)
Dilated left atrium	23 (76.7%)
Aortic stenosis	2 (6.7%)
Pericardial effusion	12 (40.0%)
Other investigations	
DPD	25 (83.3%)
Perugini grade 0	2 (6.7%)
Perugini grade I	5 (16.7%)
Perugini grade II	14 (46.7%)
Perugini grade IIII	2 (6.7%)
Bone scan	2 (6.7%)
Cardiac MRI	21 (70.0%)
Myocardial biopsy	2 (6.7%)
Treatment	
Diflunisal	2 (6.7%)
Tafamidis	10 (33.3%)
Patisiran	4 (13.3%)
Vutrisiran	5 (16.7%)
Death	19 (63.3%)

NAC = national amyloidosis centre, ATTR = transthyretin cardiac amyloidosis, DPD = 3,3-diphosphono-1,2-propanodicarboxylic acid, MRI = magnetic resonance imaging.

## Data Availability

Data for this project is available from the authors.

## Abbreviations

The following abbreviations are used in this manuscript:

ATTR-CM	transthyretin amyloid cardiomyopathy
TTR	transthyretin
SUS	secondary uses services
NAC	National Amyloidosis Centre
DPD	3,3-diphosphono-1,2-propanodicarboxylic acid
